# Case Report: A case of Rothmund–Thomson syndrome-like phenotype with an *ANAPC1* variant of uncertain significance and observed hair improvement

**DOI:** 10.3389/fmed.2026.1794483

**Published:** 2026-05-13

**Authors:** Chuhan Huang, Qingwu Liu, Dingquan Yang

**Affiliations:** 1 Beijing University of Chinese Medicine, Beijing, China; 2 Department of Dermatology, China-Japan Friendship Hospital, Beijing, China; 3 National Center of Integrated Traditional Chinese and Western Medicine, Beijing, China

**Keywords:** *ANAPC1*, case report, hair disorder, rare genodermatosis, Rothmund–Thomson syndrome, trichoscopy

## Abstract

**Background:**

Rothmund–Thomson syndrome (RTS) is a rare autosomal recessive genodermatosis typically associated with mutations in the *RECQL4* gene. However, some clinically diagnosed cases lack such variants, indicating genetic heterogeneity. *ANAPC1*, encoding a subunit of the anaphase-promoting complex (APC/C), has been implicated in RTS type 1, but its involvement in hair disorders remains unexplored.

**Case presentation:**

We report the case of a 29-year-old man who presented with lifelong sparse, fine scalp hair, bilateral malar erythema, soft fingernails, and dental anomalies (malocclusion with multiple caries). Routine laboratory tests were unremarkable except for reduced vitamins B1, B2, B6, and B9 (November 2024). Whole-exome sequencing (approximately 20,000 genes) identified a variant of uncertain significance in *ANAPC1* (NM_022662.4:c.4907T>C, p.Val1636Ala); no reportable variants were found in ACMG-recommended secondary findings genes. Combination therapy (trazodone 50 mg qn, tanshinone capsules 1 g qid, isotretinoin 20 mg qod, topical halcinonide 10 mL mixed with minoxidil 60 mL 1 mL bid, and multivitamins 2 tablets tid) was initiated in November 2024. After 6 months, follow-up trichoscopy (May 2025) showed increased hair density and shaft thickness.

**Conclusion:**

We describe a patient with a Rothmund–Thomson syndrome-like phenotype who carried a heterozygous *ANAPC1* variant of uncertain significance (VUS) and showed trichoscopic improvement after combination therapy. This singular observation hints at a possible phenotypic expansion associated with *ANAPC1* but cannot establish a new genotype–phenotype correlation. The clinical improvement underscores that symptomatic management can be beneficial in complex genodermatoses, even in the absence of a definitive molecular diagnosis. The pathogenicity of the *ANAPC1* VUS remains unconfirmed, necessitating functional validation and segregation studies in future research.

## 1 Introduction

Rothmund–Thomson syndrome (RTS) is characterized by early-onset poikiloderma, sparse hair, nail dysplasia, skeletal anomalies, and cancer predisposition, most commonly due to biallelic *RECQL4* mutations. However, approximately 20–30% of patients lack pathogenic *RECQL4* variants, suggesting genetic heterogeneity. *ANAPC1* encodes a core subunit of the anaphase-promoting complex (APC/C), a vital E3 ubiquitin ligase complex regulating mitotic exit and G1 phase entry. Biallelic intronic splicing mutations in *ANAPC1* have been reported in RTS type 1 patients (1). Given the critical role of APC/C in cell cycle regulation, *ANAPC1* dysfunction may impair hair follicle stem cell proliferation, although this potential has not been directly explored (2–4).

## 2 Case presentation

We present the case of a 29-year-old male patient born to non-consanguineous parents (father: 165 cm; mother: 171 cm), with a height of 168 cm and a weight of 65 kg. All immediate family members, including parents and a sister, had normal scalp hair.

**Figure 1:**
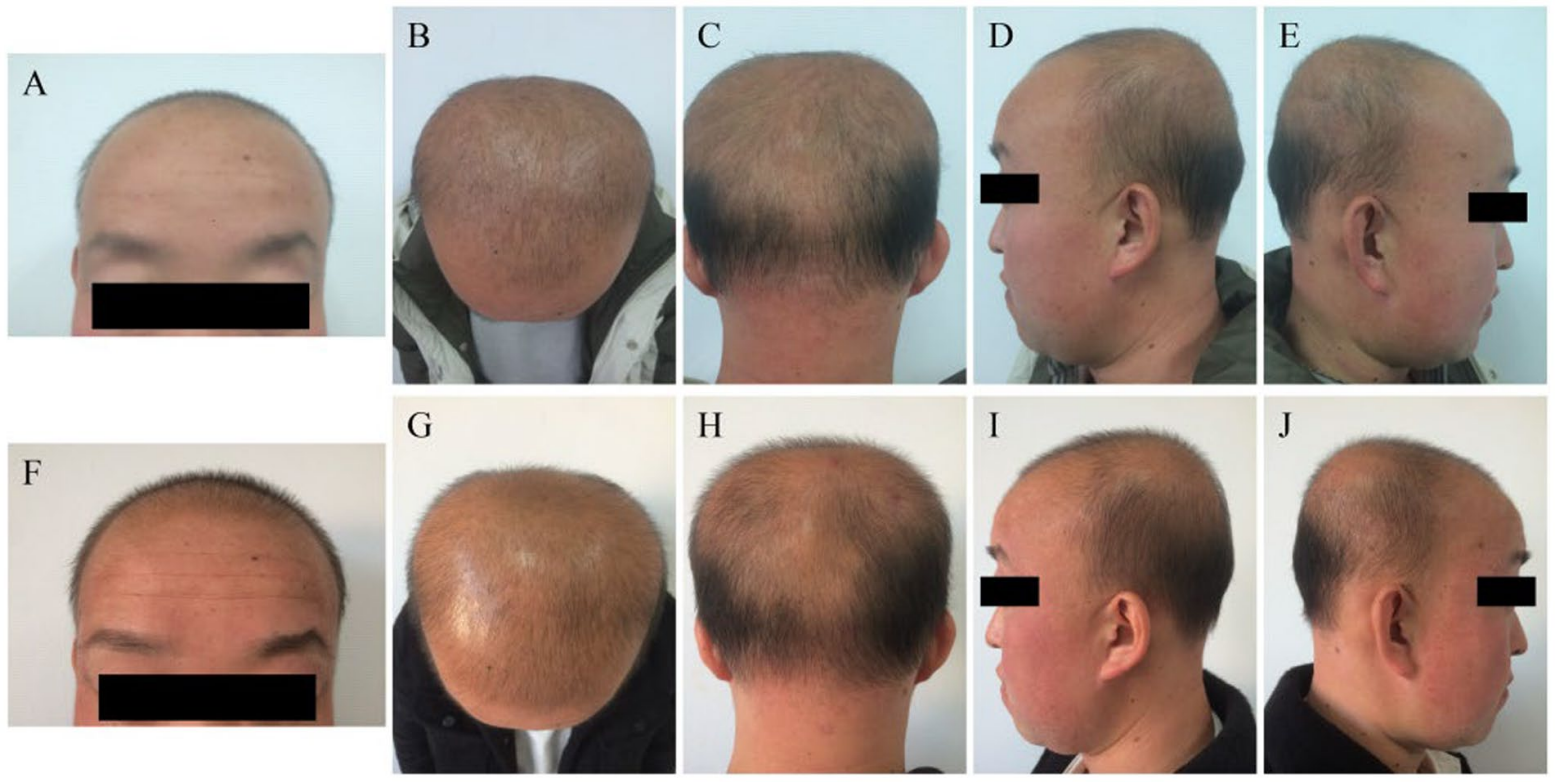
Clinical photographs of the scalp before and after 6 months of treatment. **(A–E)** Baseline images at initial presentation (November 2024) showing sparse, fine hair over the frontal, vertex, and occipital regions from multiple angles. **(F–J)** Follow-up images after 6 months of combination therapy (May 2025) showing visible improvement in hair density and coverage across the same scalp areas.

Since infancy, the patient had exhibited diffuse hypotrichosis with fine hair, most pronounced over the vertex and the occiput, accompanied by persistent bilateral malar erythema. Fingernails were soft, while skeletal and ophthalmologic examinations were unremarkable. Dental evaluation revealed malocclusion and multiple caries. Baseline clinical photographs are presented in Figures 1A–E, with 6-month follow-up images in Figures 1F–J.

In November 2024, laboratory testing showed reduced serum levels of vitamins B1, B2, B6, and B9. Liver and renal function tests, sex hormone profiles, and complete blood counts were within normal limits.

Whole-exome sequencing identified a heterozygous variant of uncertain significance (VUS) in *ANAPC1* (NM_022662.4: c.4907T>C, p.Val1636Ala; GRCh37/hg19: chr2:112542947). No pathogenic or likely pathogenic variants were found in other genes highly associated with the patient’s phenotype. Bioinformatic characterization of this variant was performed. The c.4907T>C variant is located within exon 4, which encodes a segment of the tetratricopeptide repeat (TPR) domain, a region critical for substrate recognition and co-activator binding within the APC/C complex. Multiple sequence alignment revealed that the valine residue at position 1,636 is evolutionarily conserved across vertebrate species. The variant is extremely rare in the general population, with an allele frequency of approximately 0.03% in the gnomAD database. *In silico* prediction tools yielded conflicting results: MutationTaster predicted a “disease-causing” effect, whereas Sorting Intolerant From Tolerant (SIFT) and Polymorphism Phenotyping v2 (PolyPhen-2) predicted “tolerated” and “possibly damaging,” respectively. Based on the ACMG guidelines, the variant was classified as of uncertain significance (criteria applied: PM2_Supporting, PP3_Moderate). No reportable variants were found in ACMG-recommended secondary findings genes.

Beginning in November 2024, the patient was treated with trazodone hydrochloride 50 mg nightly; tanshinone capsules 1 g four times daily; isotretinoin soft capsules 20 mg every other day; a topical mixture of halcinonide (10 mL) and minoxidil tincture (60 mL) applied at 1 mL twice daily; and multivitamin tablets, two tablets three times daily.

**Figure 2:**
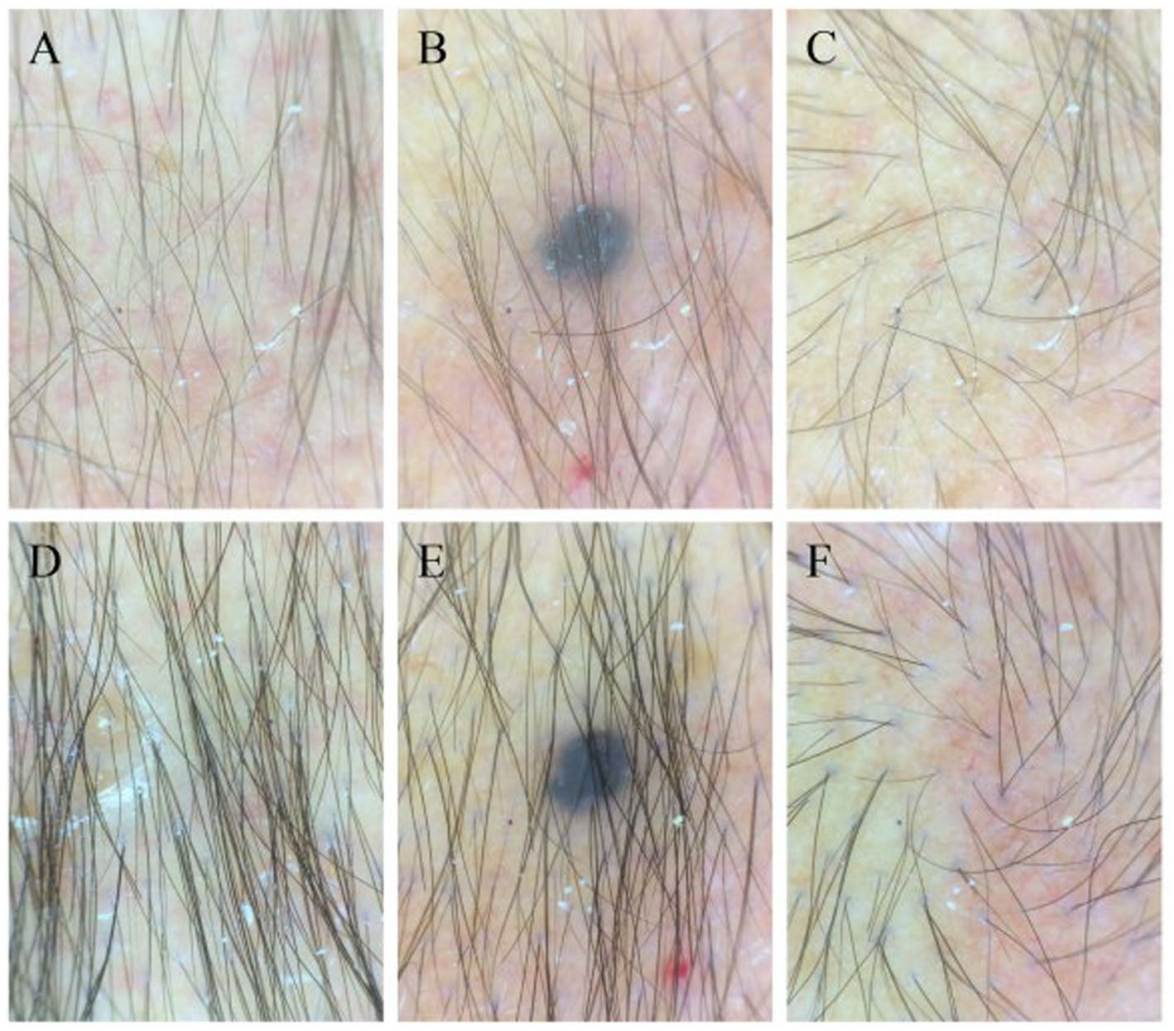
Trichoscopic findings before and after treatment. **(A–C)** Baseline trichoscopic images from the vertex region showing reduced hair density, miniaturized hair shafts, and heterogeneity in shaft thickness. **(D–F)** Follow-up trichoscopic images from the same scalp sites after 6 months of therapy showing increased hair density, thicker shafts, and reduced miniaturization.

**Figure 3:**
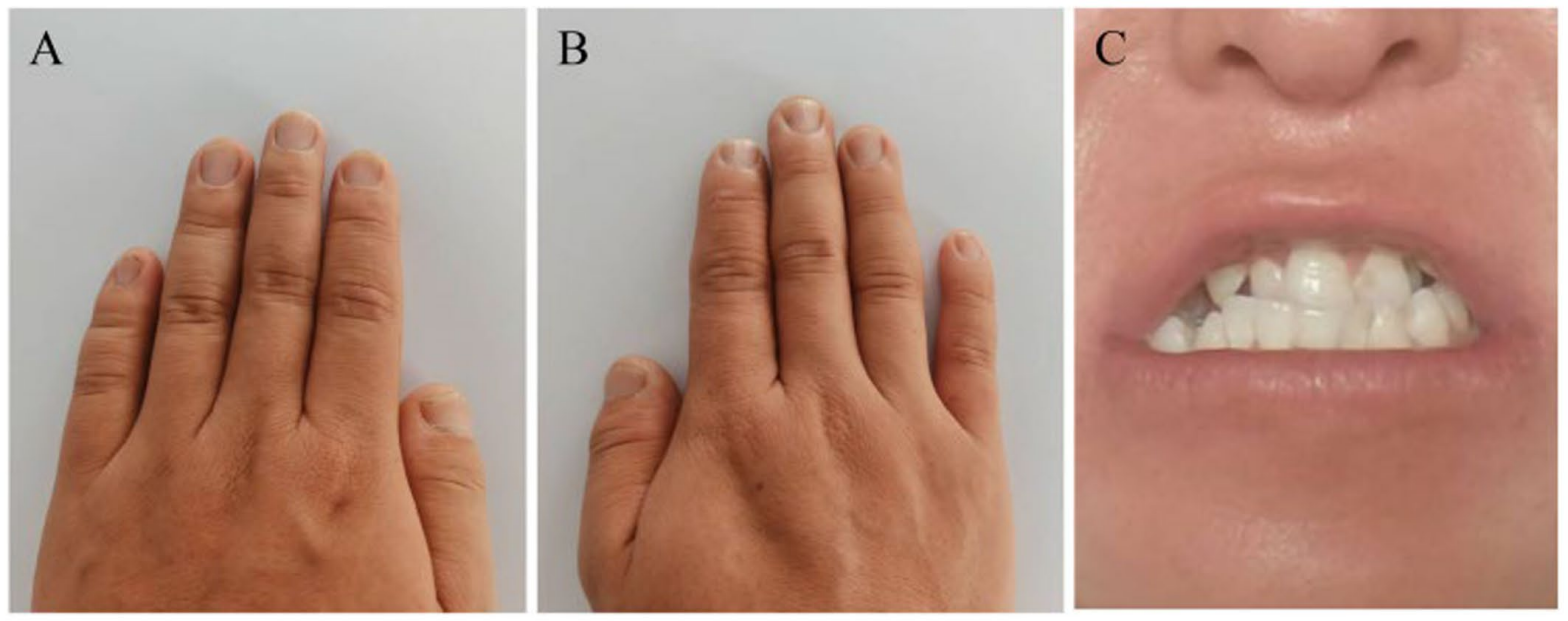
Nail and dental findings **(A,B)**. Photographs of both hands showing soft but otherwise structurally intact fingernails **(C)**. Intraoral photograph showing malocclusion and multiple dental caries.

After 6 months, follow-up trichoscopy was conducted at the same anatomically marked scalp location under identical 40 × magnification. Direct comparison of the fixed dermoscopic field showed an increased number of visible hair shafts, a greater proportion of terminal hairs, and reduced miniaturization compared with baseline (Figures 2A–C vs. Figures 2D–F). Nail and dental findings are shown in Figures 3A–C.

## 3 Discussion

RTS is classically defined by early-onset poikiloderma, sparse hair, nail abnormalities, skeletal defects, cataracts, growth retardation, and cancer predisposition, typically associated with biallelic *RECQL4* mutations (5). The patient in this report exhibited partial overlapping features, including lifelong hypotrichosis, persistent facial erythema, nail softness, and dental abnormalities (6). However, he did not exhibit skeletal anomalies, ophthalmologic involvement, growth delay, or confirmed pathogenic variants in *RECQL4* (7). Therefore, this case does not fulfill the diagnostic criteria for classical RTS and is more appropriately described as an RTS-like phenotype, reflecting partial phenotypic overlap without definitive molecular confirmation.

This RTS-like presentation with an *ANAPC1* VUS increases the possibility of alternative genetic factors contributing to the RTS spectrum. From a biological perspective, *ANAPC1* encodes a scaffolding component of the anaphase-promoting complex/cyclosome (APC/C), a multi-subunit E3 ubiquitin ligase that regulates cell cycle progression, particularly mitotic exit and G1 phase transition (8). Proper cell cycle control is essential for maintenance and activation of hair follicle stem cells, which undergo tightly regulated cycles of quiescence and proliferation during hair cycling (3, 4). Although direct evidence linking *ANAPC1* variants to hair follicle dysfunction is currently lacking, disruption of APC/C-mediated cell cycle regulation provides a biologically plausible framework through which alterations in follicular homeostasis might occur. This mechanistic consideration remains speculative and requires experimental validation.

The patient exhibited objective trichoscopic improvement after 6 months of combination therapy. The therapeutic contribution of individual components cannot be precisely delineated in this single-case context. However, the regimen was designed based on the patient’s specific presentation: the topical combination of the potent corticosteroid halcinonide with minoxidil aimed to concurrently suppress perifollicular inflammation and promote vascular dilation to prolong the anagen phase, a synergistic approach supported in the management of inflammatory alopecia (9). Isotretinoin was used for its modulating effects on follicular keratinization and potential anti-inflammatory properties (10), while tanshinone capsules, which are derived from *Salvia miltiorrhiza*, were included for their purported anti-inflammatory and microcirculatory benefits (11). Trazodone addressed comorbid scalp dysesthesia and sleep disturbance, factors known to potentially exacerbate hair loss (12). Finally, multivitamin supplementation directly corrected the identified deficiencies in vitamins B1, B2, B6, and B9, which are crucial for cellular metabolism and hair follicle function. Therefore, the clinical improvement likely resulted from the synergistic effect of mitigating inflammation, optimizing the local hair follicle environment, and correcting nutritional deficits, underscoring the rationale for a comprehensive strategy in managing complex hair disorders.

A limitation of this report is that the *ANAPC1* variant remains classified as a variant of uncertain significance, without functional validation or family segregation analysis to clarify its pathogenicity. The genetic assessment was limited to sequence-level variants detected by whole-exome sequencing; copy number variations and structural rearrangements were not specifically evaluated, and, therefore, an undetected second allele cannot be excluded. In addition, the single-case design and relatively short follow-up period limit generalizability. Nonetheless, the observed hair improvement indicates that symptomatic dermatologic management may be beneficial in similar RTS-like presentations, independent of definitive molecular confirmation.

## 4 Conclusion

We report a case of an RTS-like phenotype harboring an *ANAPC1* VUS, with observed clinical and trichoscopic hair improvement following combination therapy. This case highlights a potential novel association that warrants further investigation but does not confirm a pathogenic role for the *ANAPC1* variant. The findings underscore the value of a comprehensive dermatological workup and targeted therapy in managing complex genodermatoses, even when the underlying genetic etiology is not fully elucidated.

## Data Availability

The raw data supporting the conclusions of this article will be made available by the authors, without undue reservation.
